# Early Gut Microbiota Colonisation of Premature Infants Fed with Breastmilk or Formula with or without Probiotics: A Cohort Study

**DOI:** 10.3390/nu13114068

**Published:** 2021-11-14

**Authors:** Cheng Chi, Yali Fan, Cheng Li, Yu Li, Shan Guo, Tianhe Li, Nicholas Buys, Vicki L. Clifton, Paul B. Colditz, Chenghong Yin, Jing Sun

**Affiliations:** 1 Beijing Obstetrics and Gynecology Hospital, Capital Medical University, Beijing 100026, China; chic@mail.jnmc.edu.cn (C.C.); yalifan@mail.ccmu.edu.cn (Y.F.); guoshan@ccmu.edu.cn (S.G.); lth_felicity@ccmu.edu.cn (T.L.); 2 Menzies Health Institute Queensland, School of Medicine and Dentistry, Griffith University, Gold Coast, QLD 4215, Australia; n.buys@griffith.edu.au; 3 School of Nursing, Jining Medical University, Jining 272067, China; lc454419096@163.com; 4 School of Nursing, Weifang Medical University, Weifang 261042, China; ly992276043@163.com; 5 Faculty of Medicine, Mater Research Institute, Translational Research Institute, The University of Queensland, Brisbane, QLD 4072, Australia; vicki.clifton@mater.uq.edu.au; 6 Perinatal Research Centre, UQCCR, Faculty of Medicine, The University of Queensland, Brisbane, QLD 4072, Australia; p.colditz@uq.edu.au

**Keywords:** microbiota, probiotics, preterm infants

## Abstract

Premature infants have a fragile ecology of the gut microbiota, which is associated with many health problems and may be influenced by formula versus breast feeding. The present study investigated differences in the process of gut microbiota colonisation in preterm infants fed with breastmilk or formula with or without probiotics before 12 weeks. This cohort study recruited 138 premature infants; 31 in the breastmilk (BM) group, 59 in the probiotics formula (PF) group and 48 in the non-probiotics formula (NPF) group, according to the feeding practice they received at birth. Gut bacterial composition was identified with 16S rRNA gene sequencing in faecal samples collected at 1 week, 6 weeks and 12 weeks after birth. The alpha diversity was higher in the PF group compared to the other groups at week 1 and 6 (both *p* < 0.01) but showed no difference at week 12. The beta diversity of the three groups showed a trend towards similarity at the first two stages (*p* < 0.001 and *p* = 0.009, respectively) and finally showed no difference at week 12. Canonical redundancy analysis showed that feeding type could explain the difference in gut microbiota composition at week one and six (both *p* < 0.01). At genus level, *Bifidobacterium* was enriched in the PF group, while the *Enterococcus* and *Streptococcus* was enriched in the NPF group. In summary, formula with probiotics feeding after birth can affect gut microbiota colonisation and lead to a bacterial community with less potential pathogens.

## 1. Introduction

Preterm birth is defined as an infant born before 37 weeks’ gestation and is associated with developmental immaturity of the immune and digestive systems because of the shortened gestation at birth. The impaired intestinal barrier, immature immune system and potential development of an abnormal gut microbiome mean that preterm infants are a vulnerable population for some infectious diseases and intestinal dysbiosis, including necrotising enterocolitis (NEC) and sepsis [1,2]. The diminished gut barrier of preterm infants creates the conditions for translocation of pathogens from their gut into their bloodstream and finally the whole body, leading to some devastating infections associated with high mortality rates [3,4].

Gut microbiota is known to be associated with the health of infants, especially preterm infants. Many factors, including birth weight, gestational age, caesarean section delivery, antibiotic use and maternal pregnancy factors, can affect the colonisation process of preterm infants’ gut microbiota, and these effects may last for at least 3 years before the profile of the gut microbiota stabilises and alters to normal levels [5]. In addition, our prior studies [6,7] showed that the bacterial composition varied between infants fed with formula with or without added probiotics/probiotics, indicating that nutrition management is also critical for preterm infants in the development of a healthy gut microbiota. Fehr et al. [8] showed that mothers may transfer bacteria to the infant gut via breast feeding, and breastmilk intake may affect the offspring’s microbiota development. In this situation, it is important to determine an optimal nutrition strategy for preterm infants to promote their growth, avoid adverse events caused by disorders of gut microbiota, and finally reduce the risk of mortality.

It is well known that breastmilk feeding is the gold standard for infants’ nutritional intake in their early life, especially the first year of life [9]. Most past studies focused on the content of bioactive components, including immunoglobulins and lactoferrin, and their effect on promoting immune system growth [10,11]. In this study, we aimed to profile the early gut microbiota colonisation process of premature infants fed with breastmilk and formula with or without probiotics using a cohort study design. We compared the composition and biodiversity of gut microbiota and examined factors that influenced variation in the microbiota. Our results provide novel evidence to describe the process of preterm infants’ gut microbiota development in their early life.

## 2. Materials and Methods

### 2.1. Study Participants

The present study was conducted in accordance with the Declaration of Helsinki. The Ethics Committee of the Beijing Obstetrics and Gynecology Hospital approved the study protocol with approval ethics number 2017-KY-027-01. We informed the parents of each infant of the study details and obtained informed written consent. From 2017, a birth cohort was set up to trace the process of infants’ early gut microbiota colonisation in Beijing, China. From September 2018 to March 2019, one hundred and thirty-eight preterm infants were enrolled in this cohort study in accordance with the following inclusion criteria: infants were born with a gestation age under 37 weeks and birthweight under 2500 g and were hospitalised in the Neonatal Intensive Care Unit (NICU) following evaluation by pediatricians. Infants were excluded from the study if they had genetic defects, neural tube defects, congenital heart disease or any other surgical disease. All the premature infants received intensive care treatment as required for at least one week, during which they could not have skin-to-skin contact with their family or breastfeeding directly from their mothers. The NICU provides a stable environment with more nursing care and less pathogenic bacteria. According to the feeding practice type received, infants were divided into three groups, including the breastmilk (BM) group, probiotics formula (PF) group and non-probiotics formula (NPF) group. In the BM group, breastmilk was collected by mothers without freezing, disinfected, fed to their infants during hospitalisation, and breastfeeding was encouraged after discharge. When mothers could not provide sufficient breastmilk or chose to use formula feeding, their infants were allocated to NPF and PF groups according to the type of infant formula they chose at birth. The formula used in the PF group was supplemented with a probiotic of *Bifidobacterium lactis*.

### 2.2. Faecal Sample Collection

Faecal samples from the three groups were collected at 1 week, 6 weeks and 12 weeks after birth. We used the PSP^®^ Spin Stool DNA Plus Kit (STRATEC Biomedical AG, Birkenfeld, Germany), in which the collecting tube was pre-filled with 8 mL of DNA stabiliser solution, to collect faecal samples from soiled diapers. The DNA stabiliser solution can avoid RNA degradation for up to 3 months and enable the progress of collection and transportation at room temperature. For the discharged infants, faecal samples were delivered back to the central laboratory immediately after collection, then samples were frozen and stored at −80 °C until the next RNA extraction procedure.

### 2.3. 16S rRNA Amplicon Sequencing

RNA extraction with a bead-beater step occurred using the Spin Stool DNA Plus Kit mentioned above in accordance with the manufacturer’s instructions. We chose the V4 hypervariable region to amplify genes, which was generated using 515F (5′-GTGCCAGCMGCCGCGGTAA-3′) as a forward primer, and 806R (5′-GGACTACHVGGGTWTCTAAT-3′) as a reverse primer. The amplified gene was then sequenced and analysed to define the composition of the gut microbiota. After PCR procedure, we used AMPure XP beads to purify the products. Finally, we obtained the final amplicon libraries (about 630 bp) for sequencing. The multiplex amplified libraries were then denatured sequenced by Illumina MiSeq with 2 × 300 paired-end V4 sequencing reagents (Illumina, San Diego, CA, USA).

### 2.4. Data Analysis

According to the unique barcodes attached, we assigned the paired-end reads from the original DNA fragment to each sample and used FLASH [12] to merge them together. The QIIME (Quantitative Insights into Microbial Ecology, version 1.9.1) was then used to identify and filter out the merged sequences of poor quality (Q-score < 25). USEARCH8 [13] was used to remove chimera and conduct dereplication of the reads left. After redundancies were removed, merged sequences of high-quality were classified into unique sequences. These sequences were then assigned to the same operational taxonomic units (OTUs) if they had a similarity of more than 97%. Referring to the From each OTU, a representative sequence was chosen and assigned to different genera using the classifier approach [14] referred to the Ribosomal Database Project database [15]. Based on the genus level abundance, alpha diversity (Shannon and Simpson indices) were calculated and plotted using the Vegan package in R software (version 4.1.0) and compared using the Wilcoxon rank-sum test. In addition, based on the genus level abundance data, beta diversity was calculated using the Vegan package, and a principal component analysis (PCA) based on Bray-Curtis distances was plotted. Adonis permutational multivariate analysis of variance of Bray-Curtis distances with 9999 permutations was used to compare the microbial community structure between each of the two groups. The Benjamin–Hochberg method was used to correct *p* values for multiple testing. The Vegan package was applied to perform canonical redundancy analysis (RDA), which enabled us to define the influencing factors, which could significantly affect the gut microbial composition of each group. All these results were plotted using the ggplot2 package. We used Linear discriminant analysis (LDA) of effect size (LEfSe) to determine the most discriminant taxa between different groups.

## 3. Results

### 3.1. Clinical Characteristics of Participants

This cohort study recruited a total 138 premature infants, who were allocated to the BM group (*n* = 31), PF group (*n* = 59), or NPF group (*n* = 48), according to the feeding practices their parents chose from birth. Clinical characteristics of the participants included in the final sample for the study are displayed in Table 1. The level of gestational age and birthweight of the NPF group was significantly lower than the other two groups (both *p* < 0.01). There were no significant differences among the three groups in relation to sex, caesarean delivery rate and 1 min, 5 min, 10 min Apgar score at birth (all *p* > 0.05).

### 3.2. Longitudinal Variation of Gut Microbiota Diversity and Composition

**Figure 1:**
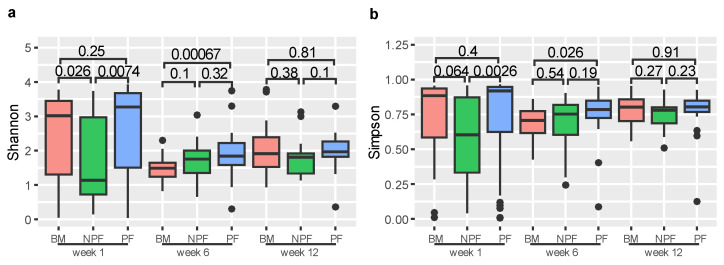
Alpha diversity of each group at three time points, measured by Shannon (**a**) and Simpson (**b**) indices. BM, breastmilk; PF, probiotics formula; NPF, non-probiotics formula.

We used Shannon and Simpson indices to measure the alpha diversity of gut microbiota (Figure 1). At week 1, the Shannon index of the NPF group was significantly lower than those in the BM and PF groups (*p* = 0.026 and *p* = 0.0074, respectively). Similarly, the Simpson index of the NPF group was significantly lower than those in the PF group (*p* = 0.0026). At week 6, the Shannon and Simpson indices of the BM group were significantly lower than the PF group (*p* = 0.00067 and *p* = 0.0026, respectively). Among the three stages, no other significant difference was observed between the groups (all *p* > 0.05).

**Figure 2:**
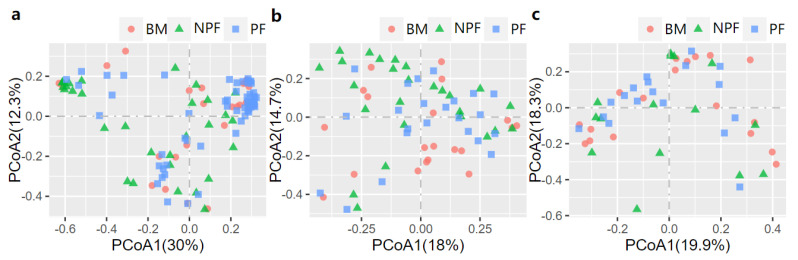
Bray−Curtis distances displayed by PCoA plots showing the profile of gut microbiota at Week 1 (**a**), Week 6 (**b**) and Week 12 (**c**). PCoA, principal coordinate analysis; BF, breastmilk feeding; FF, formula feeding.

The PCoA was then conducted to examine the spatial array of bacterial taxa in each group. The results of plotting the infant microbiota at week 1 (Figure 2a) demonstrated that the microbiota of the NPF and PF groups tended to cluster at the left and right sides of the graph, and a significant difference was observed in their beta diversity (F model = 3.494, *R*^2^ = 0.059, adonis *p* < 0.001). At week 6, differences in the bacterial taxa among the three groups were observed (F model = 2.140, *R*^2^ = 0.060, adonis *p* = 0.008), and the clustering was not obvious in the PCoA plot (Figure 2b). In addition, a significant difference in beta diversity was observed between each of the two groups (all adonis *p* < 0.05). Finally, as shown in Figure 2c, among all the three groups, no significant differences were observed at week 12 (F model = 1.155, *R*^2^ = 0.046, adonis *p* = 0.275).

**Figure 3:**
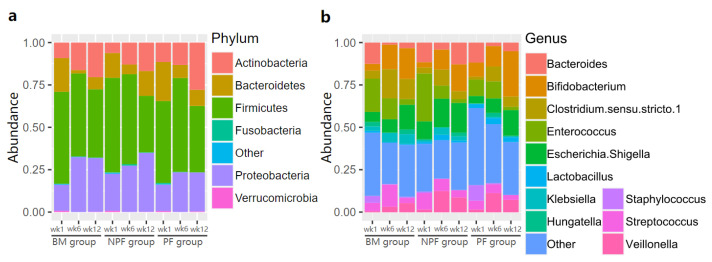
Relative abundance of gut microbiota at phylum level (**a**) and genus level (**b**). BM, breastmilk; PF, probiotics formula; NPF, non-probiotics formula.

The relative abundance of gut microbiota in the three groups was examined (Figure 3). Firmicutes was always the dominant bacteria at the phylum level in each group, making up 33.40% to 55.40% of normalised reads at each stage. At the genus level, the relative abundance of Enterococcus was greater in the NPF group than the BM (28.20% vs. 19.57%) and PF groups (9.57%). It is noted that *Bifidobacterium* was enriched in each group gradually and the PF group occupied a larger proportion than the other two groups, reaching 26.64%.

### 3.3. Differences in the Bacterial Taxa Quantified by LEfSe Analysis

**Figure 4:**
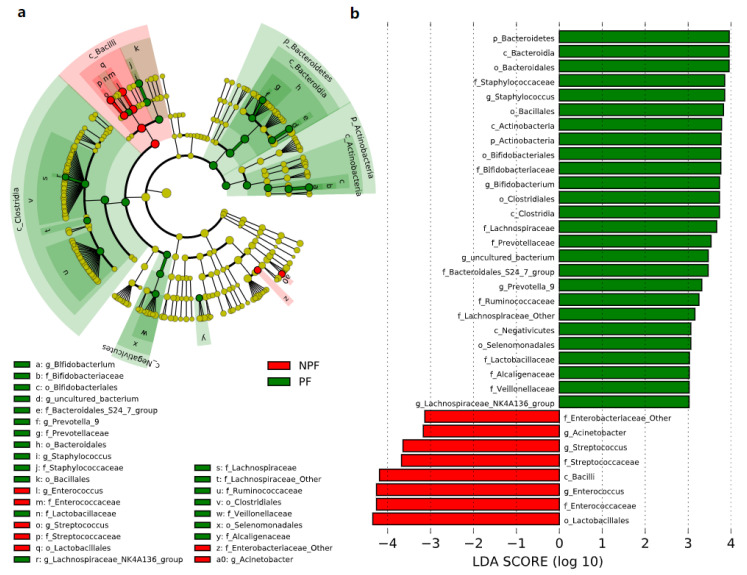
Differential bacterial taxa between the NPF and PF groups at week 1 revealed by LDA cladogram (**a**) and ranking (**b**). Only taxa with an LDA score threshold more than 3 are shown. LEfSe, linear discriminant analysis of effect size; LDA, linear discriminant analysis; PF, probiotics formula; NPF, non-probiotics formula; p, phylum; c, class; f, family; o, order; g, genus.

**Figure 5:**
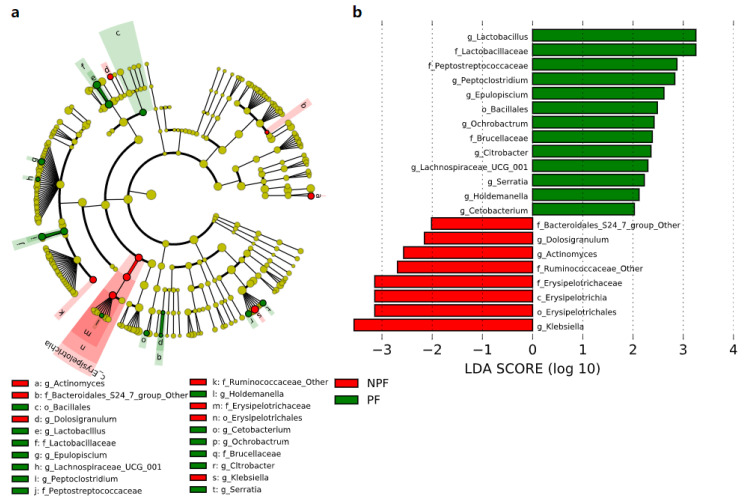
Differential bacterial taxa between the NPF and PF groups at week 6 revealed by LDA cladogram (**a**) and ranking (**b**). Only taxa with an LDA score threshold larger than 2 are shown. LEfSe, linear discriminant analysis of effect size; LDA, linear discriminant analysis; PF, probiotics formula; NPF, non-probiotics formula; p, phylum; c, class; f, family; o, order; g, genus.

We conducted LEfSe to explore the variation of the bacterial taxa among samples from different groups at week 1 and week 6. This analysis showed significant differences in beta diversity among the three groups, especially in the NPF and PF groups. At week 1 (Figure 4), the Bacteroidetes phylum (LDA = 3.957, *p* = 0.002) and Actinobacteria phylum (LDA = 3.767, *p* = 0.005) were enriched in the PF group. At the genus level, it was noteworthy that the *Bifidobacterium* genus was enriched in the PF group (LDA = 3.731, *p* < 0.001), while the *Enterococcus* and *Streptococcus* genus was enriched in the NPF group (LDA = 4.260, *p* = 0.012 and LDA = 3.642, *p* = 0.020, respectively). The result of LDA at week 6 was presented in Figure 5. Obviously, the number of discriminant taxa reduced even when we set a lower LDA value (LDA = 2.000) for discrimination, when compared with the result of week 1. We observed that the *Lactobacillus* genus was enriched in the PF group (LDA = 3.252, *p* < 0.001), while the level of *Klebsiella* genus abundance was higher in the NPF group (LDA = 3.557, *p* = 0.048).

### 3.4. Influencing Factors of Gut Microbiota Defined by RDA

**Figure 6:**
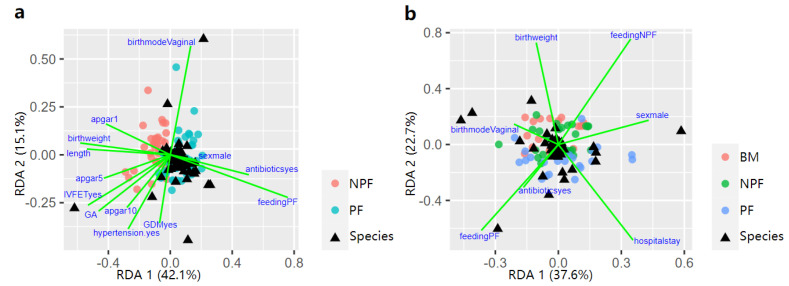
Influencing factors of the infant gut microbiota composition between NFP and PF group showed by RDA triplot at week 1 (**a**), and all the three groups at week 6 (**b**). RDA, redundancy analysis; BM, breastmilk; PF, probiotics formula; NPF, non−probiotics formula.

Possible factors that effected the process of early gut microbiota colonisation at week 1 and 6 were further explored via RDA (Figure 6). At week 1, feeding type (9.22%, *p* = 0.001), birth weight (2.11%, *p* = 0.048), gestational age (2.78%, *p* = 0.013), pregnancy-induced hypertension (2.01%, *p* = 0.039) contributed to the difference in gut microbiota spatial distribution in the NPF group and the PF group. At week 6, sex, feeding type, birth weight, gestational age, antibiotic use, and length of hospital stay were taken into consideration. Feeding type (4.16%, *p* = 0.046) and hospital stay (2.85%, *p* = 0.019) significantly explained the variation in microbiota composition among the three groups.

## 4. Discussion

We characterised the early gut microbiota profiles of 138 preterm infants fed with breastmilk or formula supplemented with probiotics or not, by collecting and analysing their faecal samples dynamically. The results of high-throughput sequencing showed the composition of gut microbiota varied among the three groups as a result of different feeding types at birth, and then showed a trend towards similarity by week 12. Further, RDA results showed that feeding type could significantly explain the variations in gut microbiota composition at week 1 and week 6.

Breastmilk contains a multitude of immunomodulating components that can reduce the risk of infectious diseases. These components, including soluble immunoglobulin A and growth factors, can promote the development and health of the intestinal barrier [16]. Breastmilk also contains specific human milk oligosaccharides (HMOs), which might protect infants with very low birth weight from infection by decreasing pathogens associated with sepsis [17]. Thus, the gut microbiota of preterm infants fed by breastmilk was also sequenced and calculated, when the gut microbiota of PF and NPF group were compared. Our previous study [18] showed that the alpha diversity of preterm infants remained at a relatively low at early stages compared with term infants, which might be associated with an increased risk of infectious diseases in premature neonates [19]. The current study found that the alpha diversity levels of the PF group was significantly higher than the other two groups at week 1 and week 6, which indicated that more species were observed in the faecal samples from infants in the PF group. This could be explained by the supplementation of *Bifidobacterium* in formula fed to the PF group. As a well-known probiotic strain, the colonisation of *Bifidobacterium* can remodel gut microbiota via its inhibiting effect on the colonisation of pathogens [6]. Our results are similar to those in a multicenter cohort study by Kurath-Koller et al. [20] that the bacterial load was increased in two weeks’ time after probiotics were used in preterm neonates. At the end of follow-up, the biodiversity of the three groups tends to be the same in our study, indicating that the species diversity of gut microbiota grows closer to each other. This process might take a period of time, which has turned out to be at least 6 weeks according to our results. Our findings confirm probiotics can be initiated early when preterm infants in the neonatal time and the diversity of bacterial load can be increased early, which might prevent neonatal morbidities.

In relation to beta diversity, significant differences were observed between the NPF and PF groups at week 1, and then among the three groups at week 6, but not at week 12, indicating that the microbiota composition of breastmilk-fed and formula-fed infants were eventually comparable. Probiotic supplements can affect the infants’ gut microbiota greatly at earlier stages. However, inappropriate use of probiotics may lead to a disorder of the intestinal microecology and cause severe infectious diseases due to the fragility of infants immune barriers at this stage. Thus, the formula added probiotics should be used with caution.

Our results also clarified the difference of bacterial abundance at genus level. Consistent with past studies that also used *Bifidobacterium lactis* as a probiotic supplement [21], at week 1, the abundance of *Bifidobacterium* was greater in the PF group. The differences between groups in bacterial composition decreased from week 1 to week 12, and fewer strains showed differences in relative abundance with time. This can be explained by the gradually colonisation of other strains, especially after discharge from the NICU, whereby infants come into contact with more strains from the external environment and daily contact with their family. Other strains that should be noted were *Enterococcus* and *Streptococcus* genus, which were enriched in the NPF group and might inhibit the normal microbiota succession [19].

Analysis of the influencing factors of the first two stages via RDA showed that the bacterial variation at week 1 and week 6 could be explained by different feeding types. Consistent with results from the present study, past studies [8,22] also reported that different feeding types can affect the gut microbial composition. The effect of feeding type on gut microbiota colonisation may be explained by the probiotics supplemented, as well as lactoferrin, HMOs and other prebiotics in breastmilk, while formula could not provide these nutritional components., Lactoferrin especially plays a critical role in remodelling gut microbiota, interacting with immune cells by binding to a variety of cytokines and causing bacteriostatic effects directly in iron-requiring pathogens [23,24,25]. Factors including birthweight and GA at birth, and pregnancy-induced hypertension status of mothers could also affect the gut microbial composition, which was confirmed in our previous studies [18]. It is noteworthy that length of hospital stay also contributed to the variation of gut microbial composition, which might be due to the difference in the environmental flora which the infants come into contact before and post discharge.

### Limitations

The main limitation of our study was the lack of analysis on functional changes associated with the microbiome profiles of each group. We used tubes filled with DNA stabiliser solution to collect the fecal samples, which enabled us to do the follow-up after infants were discharged. However, the mixture of faecal samples and DNA stabiliser solution could not be used in further functional tests. Metagenomic sequencing or more suitable methods in sample collection should be applied in future studies in preterm infants.

## 5. Conclusions

In conclusion, the current study showed the gut microbiota colonisation of premature infants under different feeding practices during the first 12 weeks after birth. Our results suggest that formula with probiotics intake could affect gut microbiota colonisation and lead to a bacterial community with less potential pathogens. Our findings confirm probiotics can be initiated early at the neonatal stage, which might prevent neonatal morbidities. The findings of the study suggest formula with probiotics is beneficial to improve preterm infants gut microbiome. Further studies using randomised controlled trials are needed to confirm our findings.

## Figures and Tables

**Table 1:** Clinical characteristics of participants.

Variables	BM (*N* = 31)	PF (*N* = 59)	NPF (*N* = 48)	*p* Value
Male, *n* (%)	13 (41.9)	27 (45.8)	21 (43.8)	0.94
Gestational age, weeks	34.23 ± 2.03	34.46 ± 3.20	32.44 ± 3.03	0.001
Birthweight, g	2059.52 ± 301.74	1861.12 ± 449.68	1810.76 ± 462.19	0.006
Cesarean delivery, *n* (%)	25 (80.6)	50 (84.7)	40 (83.3)	0.89
1 min Apgar score	9.58 ± 0.67	9.15 ± 1.30	9.71 ± 0.68	0.12
5 min Apgar score	9.94 ± 0.25	9.69 ± 0.65	9.88 ± 0.33	0.10
10 min Apgar score	9.97 ± 0.18	9.83 ± 0.42	9.88 ± 0.33	0.46
1st week Antibiotics use, *n* (%)	16 (51.6)	35 (59.3)	19 (39.6)	0.13
GDM, *n* (%)	4 (12.9)	14 (23.7)	15 (31.3)	0.45
PIH, *n* (%)	6 (19.4)	8 (13.6)	11 (22.9)	0.18

Continuous variables are represented as mean ± standard deviation; GDM, gestational diabetes mellitus; BM, breastmilk; PF, probiotics formula; NPF, non-probiotics formula; PIH, pregnancy-induced hypertension.

## Data Availability

All data generated or analyzed during this study are available from the corresponding author on reasonable request.

## References

[1] Nino D.F., Sodhi C.P., Hackam D.J. (2016). Necrotizing enterocolitis: New insights into pathogenesis and mechanisms. Nat. Rev. Gastroenterol. Hepatol..

[2] Twilhaar E.S., Wade R.M., de Kieviet J.F., van Goudoever J.B., van Elburg R.M., Oosterlaan J. (2018). Cognitive Outcomes of Children Born Extremely or Very Preterm Since the 1990s and Associated Risk Factors: A Meta-analysis and Meta-regression. JAMA Pediatr..

[3] Moschopoulos C., Kratimenos P., Koutroulis I., Shah B.V., Mowes A., Bhandari V. (2018). The Neurodevelopmental Perspective of Surgical Necrotizing Enterocolitis: The Role of the Gut-Brain Axis. Mediat. Inflamm..

[4] Wisgrill L., Wessely I., Spittler A., Forster-Waldl E., Berger A., Sadeghi K. (2018). Human lactoferrin attenuates the proinflammatory response of neonatal monocyte-derived macrophages. Clin. Exp. Immunol..

[5] Yatsunenko T., Rey F.E., Manary M.J., Trehan I., Dominguez-Bello M.G., Contreras M., Magris M., Hidalgo G., Baldassano R.N., Anokhin A.P. (2012). Human gut microbiome viewed across age and geography. Nature.

[6] Chi C., Xue Y., Liu R., Wang Y., Lv N., Zeng H., Buys N., Zhu B., Sun J., Yin C. (2020). Effects of a formula with a probiotic Bifidobacterium lactis Supplement on the gut microbiota of low birth weight infants. Eur. J. Nutr..

[7] Chi C., Buys N., Li C., Sun J., Yin C. (2019). Effects of prebiotics on sepsis, necrotizing enterocolitis, mortality, feeding intolerance, time to full enteral feeding, length of hospital stay, and stool frequency in preterm infants: A meta-analysis. Eur. J. Clin. Nutr..

[8] Fehr K., Moossavi S., Sbihi H., Boutin R.C.T., Bode L., Robertson B., Yonemitsu C., Field C.J., Becker A.B., Mandhane P.J. (2020). Breastmilk Feeding Practices Are Associated with the Co-Occurrence of Bacteria in Mothers’ Milk and the Infant Gut: The CHILD Cohort Study. Cell Host Microbe.

[9] Section on Breastfeeding (2012). Breastfeeding and the use of human milk. Pediatrics.

[10] Gridneva Z., Lai C.T., Rea A., Tie W.J., Ward L.C., Murray K., Hartmann P.E., Geddes D.T. (2020). Human milk immunomodulatory proteins are related to development of infant body composition during the first year of lactation. Pediatr. Res..

[11] Carbone F., Montecucco F., Sahebkar A. (2020). Current and emerging treatments for neonatal sepsis. Expert Opin. Pharmacother..

[12] Magoc T., Salzberg S.L. (2011). FLASH: Fast length adjustment of short reads to improve genome assemblies. Bioinformatics.

[13] Edgar R.C. (2010). Search and clustering orders of magnitude faster than BLAST. Bioinformatics.

[14] Wang Q., Garrity G.M., Tiedje J.M., Cole J.R. (2007). Naive Bayesian classifier for rapid assignment of rRNA sequences into the new bacterial taxonomy. Appl. Environ. Microbiol..

[15] Cole J.R., Wang Q., Fish J.A., Chai B., McGarrell D.M., Sun Y., Brown C.T., Porras-Alfaro A., Kuske C.R., Tiedje J.M. (2014). Ribosomal Database Project: Data and tools for high throughput rRNA analysis. Nucleic Acids Res..

[16] Nolan L.S., Parks O.B., Good M. (2019). A Review of the Immunomodulating Components of Maternal Breast Milk and Protection against Necrotizing Enterocolitis. Nutrients.

[17] Underwood M.A., Gaerlan S., De Leoz M.L., Dimapasoc L., Kalanetra K.M., Lemay D.G., German J.B., Mills D.A., Lebrilla C.B. (2015). Human milk oligosaccharides in premature infants: Absorption, excretion, and influence on the intestinal microbiota. Pediatr. Res..

[18] Chi C., Xue Y., Lv N., Hao Y., Liu R., Wang Y., Ding X., Zeng H., Li G., Shen Q. (2019). Longitudinal Gut Bacterial Colonization and Its Influencing Factors of Low Birth Weight Infants During the First 3 Months of Life. Front. Microbiol..

[19] Pammi M., Cope J., Tarr P.I., Warner B.B., Morrow A.L., Mai V., Gregory K.E., Kroll J.S., McMurtry V., Ferris M.J. (2017). Intestinal dysbiosis in preterm infants preceding necrotizing enterocolitis: A systematic review and meta-analysis. Microbiome.

[20] Kurath-Koller S., Neumann C., Moissl-Eichinger C., Kraschl R., Kanduth C., Hopfer B., Pausan M.R., Urlesberger B., Resch B. (2020). Hospital Regimens Including Probiotics Guide the Individual Development of the Gut Microbiome of Very Low Birth Weight Infants in the First Two Weeks of Life. Nutrients.

[21] Costeloe K., Hardy P., Juszczak E., Wilks M., Millar M.R., The Probiotics in Preterm Infants Study Collaborative Group (2016). *Bifidobacterium breve* BBG-001 in very preterm infants: A randomised controlled phase 3 trial. Lancet.

[22] Lyons K.E., Ryan C.A., Dempsey E.M., Ross R.P., Stanton C. (2020). Breast Milk, a Source of Beneficial Microbes and Associated Benefits for Infant Health. Nutrients.

[23] Allison L.M., Walker L.A., Sanders B.J., Yang Z., Eckert G., Gregory R.L. (2015). Effect of Human Milk and its Components on Streptococcus Mutans Biofilm Formation. J. Clin. Pediatr. Dent..

[24] Legrand D. (2016). Overview of Lactoferrin as a Natural Immune Modulator. J. Pediatr..

[25] Telang S. (2018). Lactoferrin: A Critical Player in Neonatal Host Defense. Nutrients.

